# Supplementary material for: Clinical response to azacitidine in MDS is associated with distinct DNA methylation changes in HSPCs
Source: Nat Commun. 2025 May 13;16:4451. doi: 10.1038/s41467-025-59796-x (PMC12075701; doi:10.1038/s41467-025-59796-x)
Supplement: Supplementary file 1 — Supplementary Information [file 41467_2025_59796_MOESM1_ESM.pdf]

Supplementary Information: Clinical Response to Azacitidine in MDS is Associated with Distinct DNA Methylation Changes in HSPCs

Julie A. I. Thoms et al

List of Supplementary Tables

Supplementary Table 1: Baseline demographic and disease characteristics of all participants

Supplementary Table 2: Blast and peripheral cell counts

Supplementary Table 3: Disease response summary

Supplementary Table 4: IWG2023 classifications

Supplementary Table 5: Delayed treatment cycles

Supplementary Table 6: Serious adverse events

Supplementary Table 7: Serious adverse events during the one year follow up period

Supplementary Table 8: Adverse events

Supplementary Table 9: BMT cellularity and HSPC abundance

Supplementary Table 10: Myeloid gene panel

Supplementary Data 1: Variant allele frequencies (provided as an excel file)

List of Supplementary Figures

Supplementary Figure 1: Example gating for cell cycle flow cytometry

Supplementary Figure 2: Cell cycle parameters in CD34+ HSPCs at diagnosis and across treatment cycles

Supplementary Figure 3: DAC incorporation and relative DNA methylation in PB and BM MNCs across treatment phases

Supplementary Figure 4: DAC incorporation and relative DNA methylation across treatment phases

Supplementary Figure 5: Changes in site-specific methylation over the course of AZA treatment

Supplementary Figure 6: Differences in CpG methylation and gene expression in CD34+ HSPCs at C7D1 compared to C1D1

Supplementary Figure 7: Differences in site-specific methylation in CD34+ HSPCs following six cycles of AZA treatment

Supplementary Figure 8: Differences in site-specific methylation in CD34+ HSPCs immediately following the first cycle of oral AZA

Supplementary Figure 9: Longitudinal analysis of RNA isoform expression, use of alternate transcripts, and RNA splicing.

Supplementary Figure 10: Variations in clonal composition in BM MNCs during HMA treatment

Supplementary Table 1: Baseline demographic and disease characteristics of all participants. * Cytopenias defined as haemoglobin < 100 g/L, absolute neutrophil count < 1.8×10^9/L, platelet count < 100×10^9/L.

Characteristic	MDS n= 31 (77.5%)	AML n= 6 (15%)	CMML n= 3 (7.5%)
**Age (yrs) median (min,max)**	76.2 (65.9, 87.4)	77.3 (74.9, 83.8)	73 (73, 82.1)
**Sex n(%) Female**	9 (22.5)	2 (5)	1 (2.5)
**Male**	22 (55)	4 (10)	2 (5)
**ECOG PS n (%)**			
0	17 (42.5)	1 (2.5)	1 (2.5)
1	10 (25)	4 (10)	2 (5)
2	4 (10)	1 (2.5)	0
**BMA blast count % median (min,max)**	12 (1, 20)	22.5 (20, 30)	11 (10, 18)
**IPSS risk group n= (%)**			
Low	0		
Int-1	0		
Int-2 (1.5-2)	22 (71)		
High risk (≥ 2.5)	9 (29)		
**IPSS-R risk group n= (%)**			
Very low	0		
Low	1 (3)		
Intermediate	3 (10)		
High	15 (48)		
Very high	12 (39)		
**Karyotype n= (%)**			
Normal, Y-, 5q-, 20q-	13 (42)		
Abnormal chromosome 7 or 3 or more abnormalities	13 (42)		
All other cytogenic abnormalities	5 (16)		
**Cytopenia of 2 or 3 cell types* n= (%)**			
No cytopenia or cytopenia of 1 cell type	6 (19)		
Cytopenia of 2 or 3 cell types	25 (81)		
**ELN risk stratification n= (%)**			
Favourable		1 (17)	
Intermediate		3 (50)	
Adverse		2 (33)	
**WHO subtype**			
CMML-1 blasts			0 (0)
CMML-2 blasts			3 (100)
**Hematology**			
Hemoglobin median (min,max) g/L	96 (73, 132)	88 (79, 121)	106 (95, 124)
ANC median (min,max) x 10^9/L	0.8 (0.2, 26.7)	1.7 (0.1, 6.7)	8.9 (4.3, 50.3)
WCC median (min,max) x 10^9/L	2.3 (0.9, 74.1)	3.4 (0.7, 23)	14.8 (8.5, 81.1)
Platelet median (min,max) x 10^9/L	61 (9, 438)	43.5 (28, 320)	40 (23, 99)

Supplementary Table 2: Blast and peripheral cell counts at screening and clinical assessment points (C7D1 – following 6 cycles of injected AZA; endpoint – following 6 cycles of oral AZA or at progression)

Patient	Haemoglobin (g/L)			Neutrophils (x10^9/L			Platelets (x10^9/L			BMA_blasts (%)		
	Screen	C7D1	End	Screen	C7D1	End	Screen	C7D1	End	Screen	C7D1	End
**P01**	111	105	92	0.4	1.1	0.8	54	47	23	12	5	2
**P02**	80	76	73	0.8	1	0.4	36	46	11	15	7	12
**P03**	96	120	106	2.9	3.8	5	89	143	166	2	2	1
**P04**	89	123	68	2.2	1.1	2.1	37	201	48	21	0	61
**P05**	132	133	88	2.5	1.2	0.9	74	106	52	15	4	9
**P06**	91	98	70	0.6	0.4	0.2	23	95	31	1	0	3
**P07**	98	99	140	0.4	0.2	0	36	28	63	12	7	1
**P08**	101	81	73	1.2	0.5	0.2	110	35	23	15	8	35
**P09**	91	75	96	0.2	0.2	0.1	27	51	79	7	6	1
**P10**	108	131	83	1.6	1	0.4	148	102	221	18	20	7
**P11**	114	106	122	1	0.2	2.9	225	317	221	15	2	1
**P12**	104	112	104	0.9	2.9	2	87	114	118	11	1	1
**P13**	98	110	111	0.9	0.9	1.9	102	282	93	18	16	
**P14**	95	110	90	1.8	1.3	1.4	176	334	107	21	0	2
**P15**	130	136		0.7	0.6		59	106		15	9	
**P16**	124	116		8.9	3.6		40	65		10	9	
**P17**	129	134	105	1.2	1.1	0.4	124	217	73	13	3	15
**P18**	91	133	84	3.2	4.3	1.5	72	144	8	5	3	31
**P19**	95	98		50.3	1		99	84		18	8	
**P20**	76	139		1.8	2.1		44	53		1	3	
**P21**	79	122		0.6	0.7		32	196		5	4	
**P22**	87	95		0.1	0.1		50	28		20	38	
**P23**	86			1.1			163			10		
**P24**	121		112	1		0.3	32		37	26		40
**P25**	85	100		1.6	5.2		28	14		24	0	
**P26**	104			0.5			99			11		
**P27**	75		75	3.9		4.9	64		13	16		59
**P28**	98		87	0.4		0	102		18	12		35
**P29**	108			0.6			53			20		
**P30**	73			0.4			29			6		
**P31**	97			1.7			9			14		
**P32**	92			1.1			61			4		
**P33**	90			26.7			438			14		
**P34**	83			0.3			28			1		
**P35**	87			0.4			57			5		
**P36**	96			0.5			22			13		
**P37**	79			6.7			320			30		
**P38**	77		76	0.4		0.4	144		29	11		62
**P39**	96		84	0.4		0.2	34		6	14		52
**P40**	106		99	4.3		5	23		16	11		64

Supplementary Table 3: Disease response summary. Shown are the number of participants achieving the indicated IWG response at end of injected phase (C6) and end of oral phase (C12) and a quantification of best response for each participant who completed the injection phase. Disease progression reported at timepoints other than IWG assessment at C6 and C12 is detailed in Figure 1C.

	**IWG response**	**C6D29**	**C12D29**	**Best response**
Responder	Complete remission (CR)	7	2	8
	Partial remission (PR)	0	0	0
	Marrow CR	3	4	4
	Hematological improvement (HI)	6	1	6
Non-responder	Stable disease (SD)	5	2	3
	Failure	1	0	1
	Relapse after CR or PR	0	1	0
	Disease progression	2	1	2
Totals		24	11	24

Supplementary Table 4: IWG2023 classifications. Compared to IWG2006, classification of one patient (*; P10) changed from non-responder to responder at C12D29.

Patient	Injection phase		Oral phase	
	**IWG 2023**	**Response**	**IWG 2023**	**Response**
P01	CRbi	Responder	CRh	Responder
P02	No response	Non-responder		
P03	CR	Responder	CR	Responder
P04	CR	Responder	PD	Non-responder
P05	CR	Responder	Disease relapse	Non-responder
P06	HI-P	Responder	No response	Non-responder
P07	No response	Non-responder	CRuni	Responder
P08	No response	Non-responder		
P09	No response	Non-responder	CRh	Responder
P10	HI-E	Responder	HI	Responder *
P11	CRbi	Responder	CR	Responder
P12	CR	Responder	CR	Responder
P13	HI	Responder	HI	Responder
P14	CR	Responder		
P15	HI-P	Responder		
P16	No response	Non-responder		
P17	CR	Responder		
P18	CR	Responder		
P19	HI	Responder		
P20	HI-E	Responder		
P21	CRbi	Responder		
P22	No response	Non-responder		
P24	No response	Non-responder		
P25	No response	Non-responder		

Supplementary Table 5: Delayed treatment cycles. Shown are duration and reasons for cycle delays of greater than 3 days.

Patient	Cycle	Duration (days)	Reason
P01	7	7	Participant holidays/work
	8	7	Haematological toxicity
	9	14	Haematological toxicity
	10	7	Haematological toxicity
	11	7	Haematological toxicity
	12	7	Haematological toxicity
P02	4	21	Non-haematological toxicity
	6	28	SAE/AE followed by suspected disease progression
P03	7	7	Unable to obtain PD bloods
P04	1	14	SAE
P06	2	11	SAE in previous cycle
P07	9	11	Participant holidays/work
P08	4	28	Participant holidays/work
	7	21	Haematological toxicity
	10	10	Haematological toxicity
P09	11	7	SAE
P10	2	7	Haematological toxicity
P14	5	14	Xmas closure
	10	14	Suspected disease progression
P18	7	4	Haematological toxicity
P19	2	7	Brief hospitalisation for Colitis (Grade 2)
P23	3	21	Non-haematological toxicity
	5	14	Haematological toxicity
P24	3	7	SAE
	4	8	Haematological toxicity
	5	6	Chest pain - hospitalisation
P32	2	7	Non-haematological toxicity

Supplementary Table 6: Serious adverse events (SAE). Table indicates the number of participants experiencing each SAE in each treatment cycle.

SAE event	Cycle - injection phase							Cycle - oral phase						
	**1**	**2**	**3**	**4**	**5**	**6**	**Total**	**7**	**8**	**9**	**10**	**11**	**12**	**Total**
febrile neutropenia	3	3	4	2			12					1		1
anaemia	1	1	1				3			1	1			2
pyrexia				1		2	3	1						1
thrombocytopenia	2			1			3	1						1
sepsis			1		1		2							0
mouth haemorrhage		1	1				2							0
musculoskeletal pain			1	1			2							0
upper respiratory tract infection	1		1				2							0
rectal haemorrhage				1			1			1			1	2
diarrhoea				1			1				1			1
neutropenia				1			1				1			1
abdominal pain	1						1							0
acute kidney injury			1				1							0
acute pulmonary oedema				1			1							0
anal fissure	1						1							0
cellulitis	1						1							0
cerebrovascular accident	1						1							0
coronary artery disease				1			1							0
haemolytic anaemia	1						1							0
haemorrhoid infection			1				1							0
hepatic failure			1				1							0
hyponatremia		1					1							0
intra-abdominal haematoma	1						1							0
jejunal perforation				1			1							0
joint swelling			1				1							0
nasal vestibulitis			1				1							0
oedema peripheral	1						1							0
pain		1					1							0
parotid gland enlargement				1			1							0
pericardial effusion			1				1							0
pneumonia			1				1							0
septic shock	1						1							0
skin infection		1					1							0
soft tissue infection					1		1							0
transitional cell carcinoma		1					1							0
upper GI haemorrhage				1			1							0
urinary tract infection					1		1							0
uterine cancer						1	1							0
myocardial infarction							0			2				2
cardiac failure acute							0						1	1
escherichia sepsis							0				1			1
lung infection							0						1	1
prostate infection							0			1				1

Supplementary Table 7: Serious adverse events (SAE) during the one year follow up period. Table indicates the number of participants experiencing each SAE. Only SAE resulting in death were recorded during the follow up period.

SAE event	Total
acute myeloid leukemia	8
myelodysplastic syndrome	3
multiple organ dysfunction syndrome	2
bladder cancer	1
cardiac arrest	1
cerebral haemorrhage	1
ischaemic gastritis	1
sepsis	1
subarachnoid haemorrhage	1

Supplementary Table 8: Adverse events (AE). Grade 4 haematological and Grade 3 non-haematological AEs. Table indicates the number of participants experiencing each AE in each treatment cycle.

AE grade	AE event	Cycle - injection phase							Cycle - oral phase						
		1	2	3	4	5	6	Uniq PIDs	7	8	9	10	11	12	Uniq PIDs
HAEM AE Grade 4	neutropenia	7	9	8	6	4	4	16	5	3	4	4	3	3	12
	thrombocytopenia	11	10	6	4	4	3	15	3	1	2	3	2	2	9
	white blood cell count decreased	1	2	2	2	0	0	3	0	0	0	1	0	1	2
Non HAEM AE Grade 3	abdominal pain								0	0	1	0	0	0	1
	anxiety								0	0	1	0	0	0	1
	ascites	0	1	0	0	0	0	1							
	blood creatinine increased	0	0	1	0	0	0	1							
	chronic obstructive pulmonary disease	0	0	0	1	1	0	1							
	diarrhoea								1	1	1	1	0	0	2
	epistaxis	0	1	0	0	0	0	1							
	haematuria								0	1	0	0	0	0	1
	hyperbilirubinaemia	0	0	2	0	0	0	2							
	hyperglycaemia	0	0	0	1	0	0	1							
	hypokalaemia	0	0	0	1	0	0	1							
	hyponatremia	0	0	1	0	0	0	1							
	nausea								1	2	0	1	0	0	3
	sebaceous carcinoma	0	0	0	0	1	0	1							
	upper gastrointestinal haemorrhage	0	0	0	0	1	0	1							
	urinary tract infection	0	1	0	0	0	0	1							
	vomiting								0	0	0	1	0	0	1
	sepsis								0	0	1	0	0	0	1
**Total unique PIDs**		**16**	**19**	**15**	**9**	**8**	**7**	**28**	**8**	**6**	**7**	**7**	**3**	**4**	**19**

Supplementary Table 9: Cellularity of bone marrow trephines (BMT), relative abundance of HSPCs in bone marrow aspirate (BMA), and proportion of blasts in BMA at diagnosis for all patients with clinical outcome data available.

Patient	Diagnosis	HSPC (% of MNCs)	BMT cellularity	BMA blast %
P01	MDS	9.4	hypercellular (3)	12
P02	MDS	5.9	marked hypercellular (4)	15
P03	MDS	1.2	hypercellular (3)	2
P04	AML	19.5	marked hypercellular (4)	21
P05	MDS	1.9	hypercellular (3)	15
P07	MDS	23.6	marked hypercellular (4)	12
P08	MDS	9.9	hypercellular (3)	15
P09	MDS	18.3	hypercellular (3)	7
P10	MDS	23.4	hypocellular (1)	18
P11	MDS	17.0	hypercellular (3)	15
P12	MDS	5.2	hypercellular (3)	11
P13	MDS	26.5	hypercellular (3)	18
P14	AML	2.8	normocellular (2)	21
P15	MDS	26.5	normocellular (2)	15
P16	CMML	13.6	hypercellular (3)	10
P17	MDS	14.1	hypocellular (1)	13
P18	MDS	6.9	marked hypercellular (4)	5
P19	CMML	0.8	hypercellular (3)	18
P20	MDS	2.9	hypercellular (3)	1
P21	MDS	23.7	hypercellular (3)	5
P22	AML	33.0	marked hypercellular (4)	20
P24	AML	40.6	hypercellular (3)	26
P25	AML	70.7	marked hypercellular (4)	24
P27	MDS	21.6	marked hypercellular (4)	16
P28	MDS	21.9	hypercellular (3)	12
P38	MDS	18.7		11

Supplementary Table 10: Myeloid gene panel

Gene Names		
ABCB1	*GATA1*	*PTEN*
ABCG2	*GATA2*	*PTPN11*
ABL1	*GATA3*	*PTPRT*
ALAS2	*GNAS*	*RAD21*
ANKRD26	*GNB1*	*RAF1*
ASXL1	*HIST1H1E*	*RPL11*
ATM	*HRAS*	*RPL35A*
ATR	*IDH1*	*RPL5*
AXL	*IDH2*	*RTEL1*
BCOR	*IKZF1*	*RUNX1*
BCORL1	*IL7R*	*SAMD9*
BRAF	*JAK1*	*SAMD9L*
BRCA1	*JAK2*	*SAMHD1*
BRCA2	*JAK3*	*SBDS*
BRD4	*KDM6A*	*SETBP1*
CALR	*KIT*	*SF1*
CBL	*KMT2A*	*SF3A1*
CCND2	*KRAS*	*SF3B1*
CDA	*LIG4*	*SMARCA2*
CDKN2A	*MECOM*	*SMC1A*
CEBPA	*MED12*	*SMC3*
CSF1R	*MPL*	*SRP72*
CSF3R	*MYD88*	*SRSF2*
CTC1	*MYSM1*	*STAG2*
DAXX	*NOTCH1*	*STIM1*
DCK	*NPM1*	*SYK*
DCLK1	*NRAS*	*TERC*
DDX41	*PARN*	*TERT*
DIS3	*PDGFRA*	*TET2*
DKC1	*PHF6*	*TINF2*
DNAJC21	*PIK3CD*	*TP53*
DNMT3A	*PIK3CG*	*TYK2*
ERCC6L2	*PLCG2*	*U2AF1*
ETV6	*PPM1D*	*U2AF2*
EZH2	*PRF1*	*WAC*
FBXW7	*PRKCB*	*WT1*
FLT3	*PRKD3*	*ZRSR2*

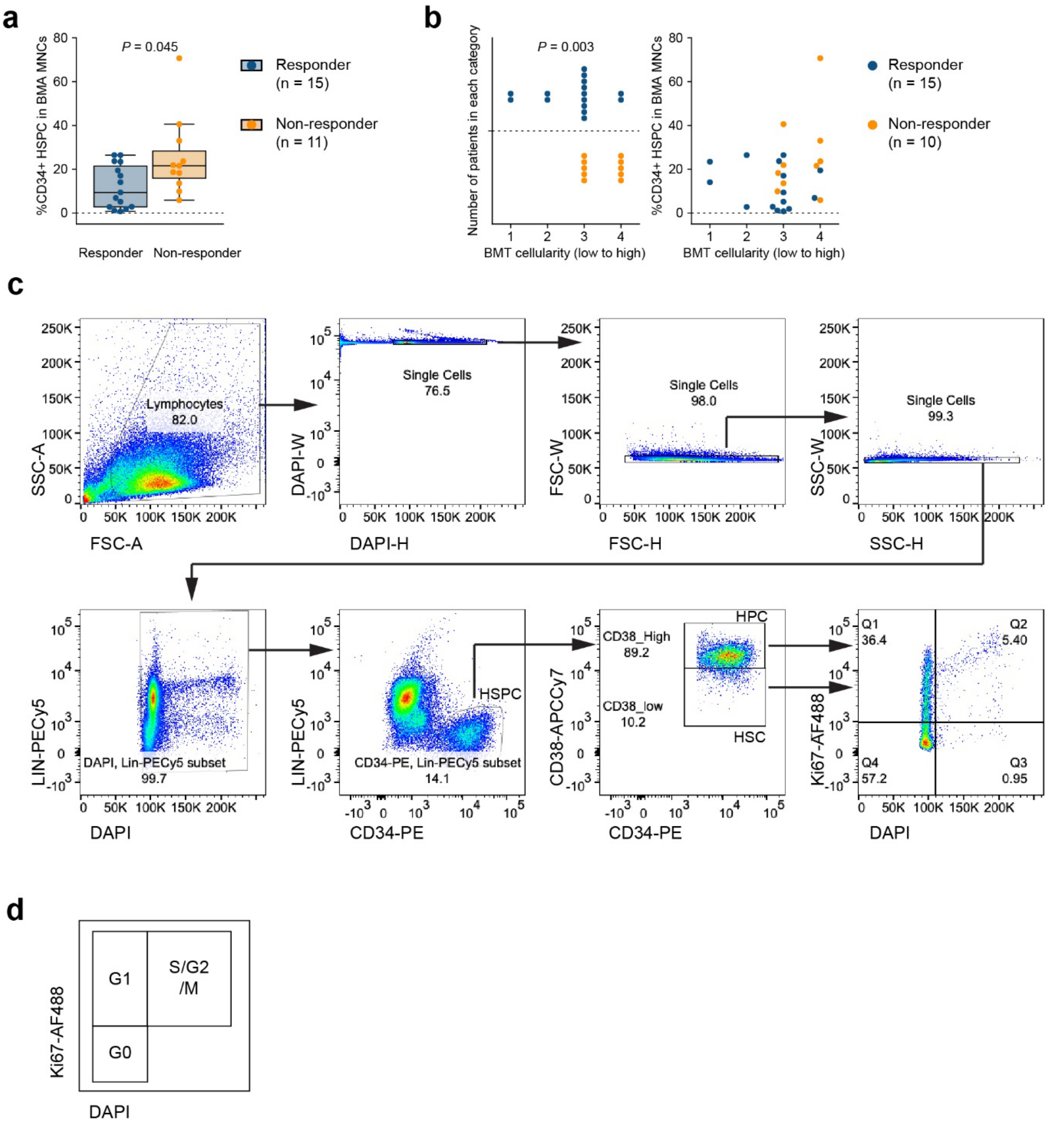

Supplementary Figure 1

Supplementary Figure 1: HSPC abundance and cellularity in bone marrow and gating of stem and progenitor cells for cell cycle flow cytometry.

(a) Proportion of BM-MNCs which are immunophenotypically HSPCs at C1D1, split by response at C7D1. P = 0.045, unequal variances two-sample t-test (two-sided), n=26. **(b)** Left: Distribution of bone marrow cellularity scores in responders and non-responders at C1D1. 1 – hypocellular, 2 – normocellular, 3 – hypercellular, 4 – markedly hypercellular. The proportion of responders decreases as cellularity increases (gamma test, gamma = -0.7895, p = 0.003) Right: Proportion of BM-MNCs which are immunophenotypically HSPCs at C1D1, split by bone marrow cellularity scores. A linear model fit to %CD34+ HSPC by responders (y vs n), cellularity and their interaction term indicates no clear relationship between BMT cellularity and %CD34+ HSPC in BMA. **(c)** Full gating strategy for analysis of cell cycle parameters in $CD34^{+}$ HSCs and HPCs. **(d)** Assignment of cell cycle phase based on final Ki67 vs DAPI gate.

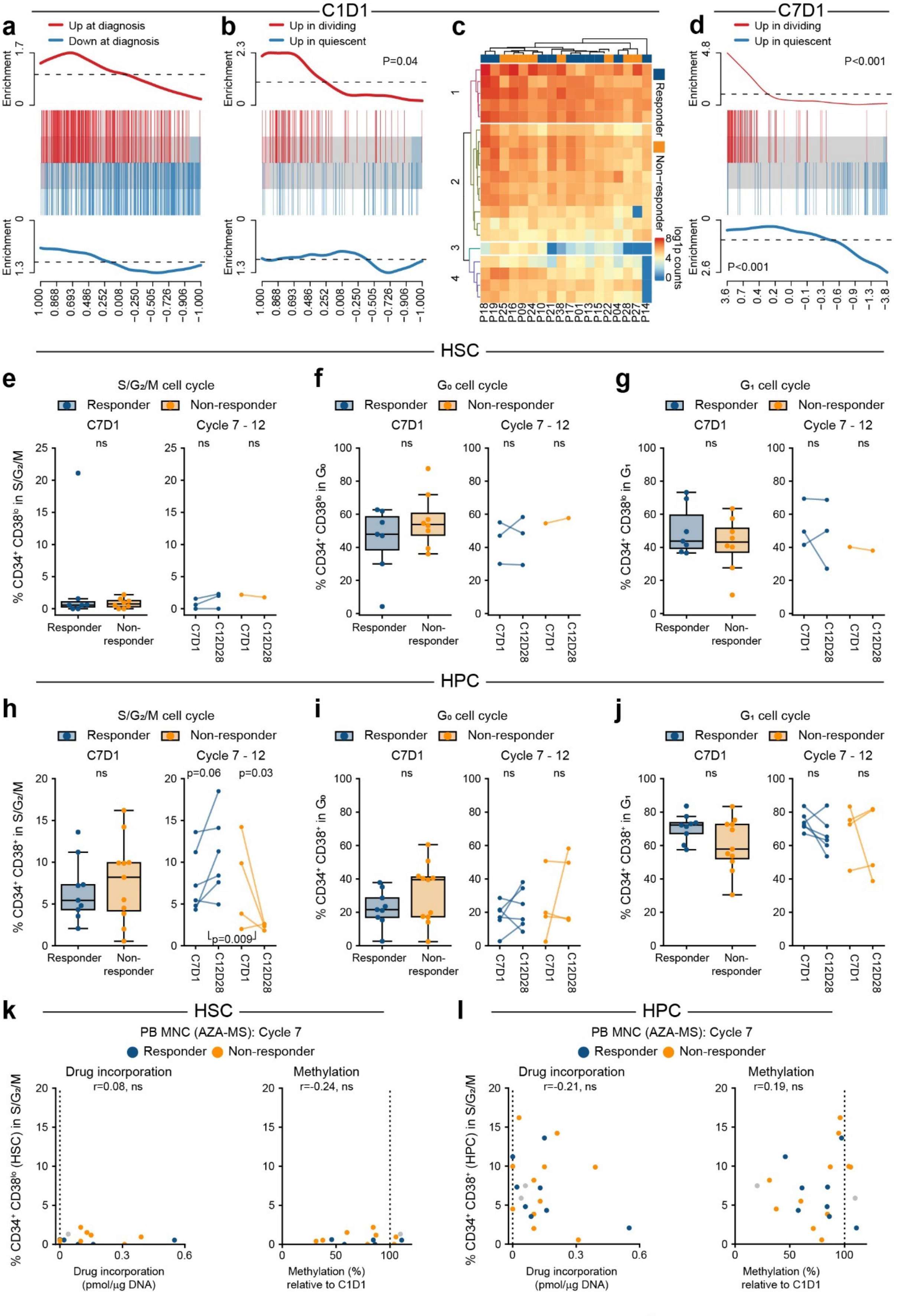

Supplementary Figure 2

Supplementary Figure 2: Cell cycle parameters in CD34+ HSPCs at diagnosis and across treatment cycles

(a-d) Gene expression at diagnosis and C7D1. P values refer to FGSEA analysis, n=18 patients. (**a**) Genes associated with patient response to AZA [1]. Red: upregulated in AZA responders. P=0.93. Blue: downregulated AZA responders. P=0.98. **(b)** Genes associated with proliferation or quiescence in $CD34^+$ cells [2]. Red: upregulated in proliferating $CD34^+$ cells. P=0.04. Blue: upregulated in quiescent $CD34^+$ cells. P=0.25. **(c)** Baseline expression of 20 genes previously used to predict patient response [1]. **(d)** Responders at C7D1 vs C1D1 showing enrichment of genes associated with CC in $CD34^+$ cells [2]. **(e-g)** Percentage of HSCs in **(e)** $S/G_2/M$, **(f)** G_0, **(g)** G_1 cell cycle phases. Samples with <50 immunophenotypic HSCs not shown. **(e-g)** Left: percentage of cells in specified cell cycle phase at C7D1, coloured by clinical outcome at end of oral phase. Responders: n=7, non-responders: n=8. P values are as indicated, ns denotes P>0.05, Welch's t-test. Right: Percentage of cells in specified cell cycle phase at C7D1 and end (C12D28) of the oral phase. Lines indicate paired samples, only patients with data at C7 and C12 are shown. Responders: n=3, non-responders n=1. P values are indicated, ns denotes P>0.05, two-sided linear mixed model. **(h-j)** Percentage of HPCs in **(h)** $S/G_2/M$, **(i)** G_0, **(j)** G_1 CC phases. **(h-j)** Left: percentage of cells in specified cell cycle phase at C7D1, coloured by clinical outcome at end of oral phase. Responders: n=6 non-responders n=4. P values as indicated, ns denotes P>0.05, Welch's t-test. Right: Percentage of cells in specified CC phase at C7D1 and end (C12D28) of the oral phase. Lines indicate paired samples, only patients with data at C7 and C12 are shown. Responders: n=6, non-responders n=4. P values are indicated, ns denotes P>0.05, two-sided linear mixed model. **(k-l)** Relationship between cell cycle status and drug incorporation and DNA demethylation during cycle 7 in **(k)** HSCs and **(l)** HPCs. **(k-l)** Percentage of cells actively cycling ($S/G_2/M$) at C7D1 compared to maximum drug incorporation (left) and minimum relative DNA methylation (right) in peripheral blood during

the same cycle. Dashed lines indicate baseline, gray dots show patients with no response data. r = two-sided spearman correlation coefficient.

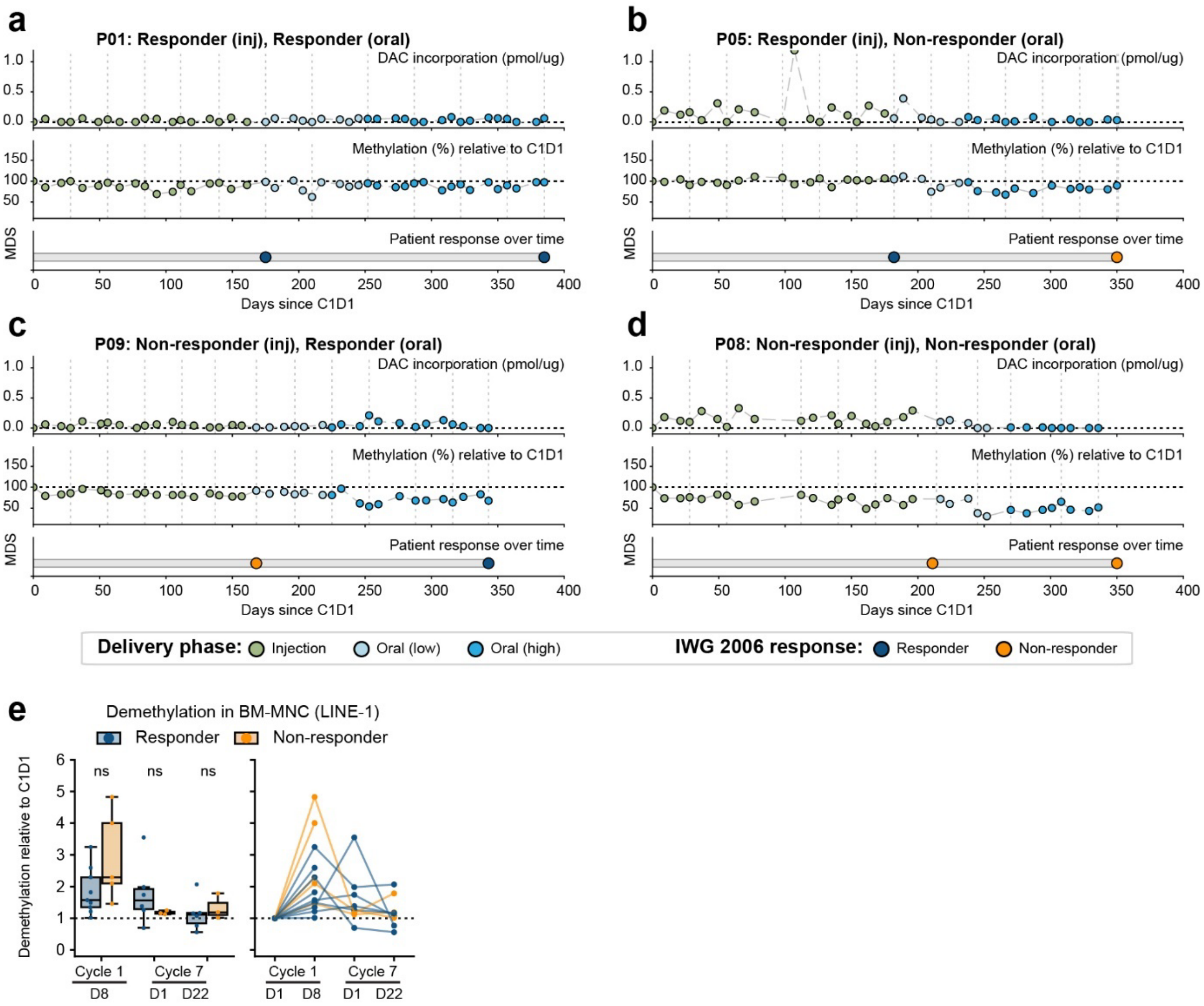

Supplementary Figure 3

Supplementary Figure 3: DAC incorporation and relative DNA methylation in PB and BM MNCs across treatment phases.

(a-d) Full kinetics of DAC incorporation and relative methylation across the complete treatment timeline for 4 participants with differing patterns of clinical response. Circles show response at IWG assessment or progression timepoints (blue – responder, orange – non-responder). Vertical lines indicate start of each treatment cycle. **(e)** Relative LINE-1 DNA demethylation in bone marrow (BM) mononuclear cells (MNC) compared to pre-treatment (n=14 patients, responder: 9, non-responder: 5). Left: aggregate data at each timepoint. Right: Longitudinal methylation changes. Statistical comparisons for panels were performed using two-sided ttest_ind (scipy.stats).

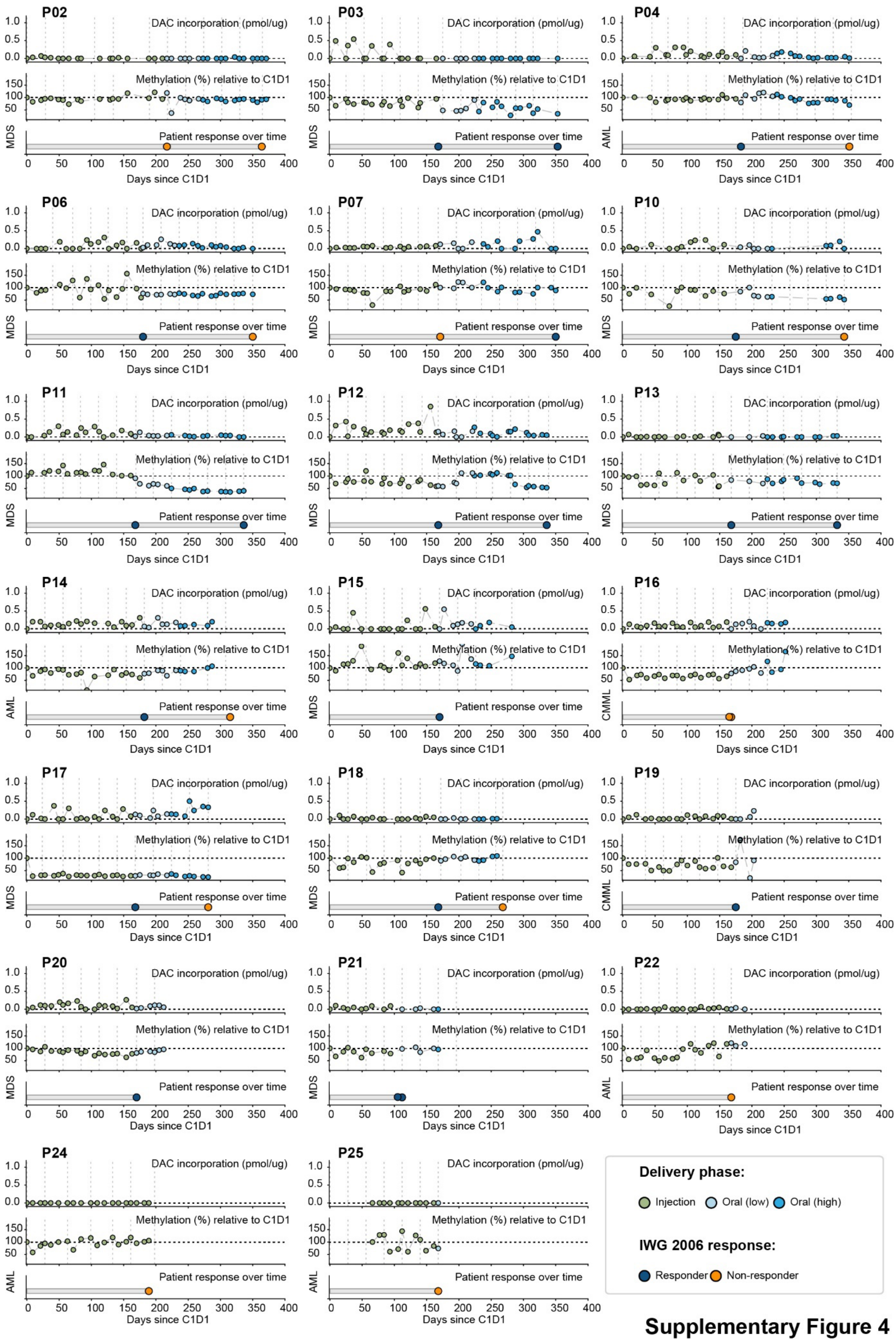

Supplementary Figure 4

Supplementary Figure 4: DAC incorporation and relative DNA methylation across treatment phases.

Full kinetics of DAC incorporation and relative methylation across the complete treatment timeline for 20 participants with clinical response assessment at the end of cycle 6. Circles show response at IWG assessment or progression timepoints (blue – responder, orange – non-responder). Vertical lines indicate start of each treatment cycle.

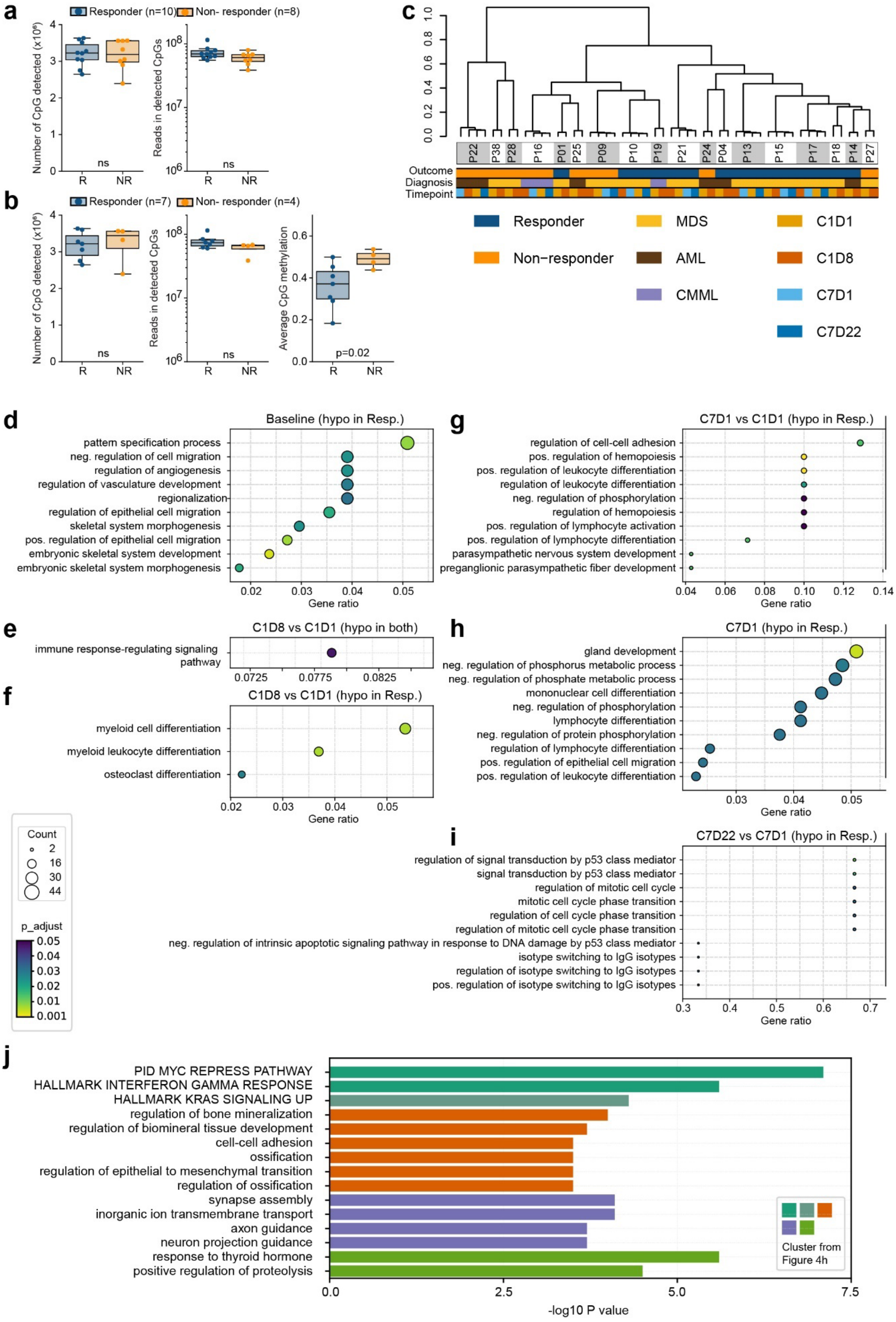

Supplementary Figure 5

Supplementary Figure 5: Changes in site-specific methylation over the course of AZA treatment

(a-c) Baseline RRBS data characteristics. **(a)** All samples. Left: Total number of CpG detected at C1D1. Right: Total number of reads covering detected CpGs at C1D1. Boxplots throughout this figure show center=median, box=interquartile range (IQR), whiskers=furthest point within 1.5xIQR, outliers=points >1.5xIQR. Statistical comparison is unpaired two-sided Welch's t-test, ns indicates $P > 0.05$. **(b)** MDS samples only. Left: Total number of CpG detected at C1D1, Centre: Total number of reads covering detected CpGs at C1D1. Right: Average methylation at CpG sites at baseline (C1D1). Statistical comparison is unpaired two-sided Welch's t-test, ns indicates $P > 0.05$. **(c)** Hierarchical clustering of all samples based on the most variable CpGs (top 50% based on standard deviation). **(d-i)** Gene ontology analysis for genes associated with differentially methylated CpGs, statistics derived from clusterProfiler[3] using GO pathways[4]. **(d)** Pathways enriched for genes associated with differentially methylated CpGs hypomethylated in responders compared to non-responders at baseline C1D1. **(e)** Pathways enriched for genes associated with differentially methylated CpGs hypomethylated in both responders and non-responders at C1D8 vs C1D1. **(f)** Pathways enriched for genes uniquely associated with differentially methylated CpGs hypomethylated in responders at C1D8 vs C1D1. **(g)** Pathways enriched for genes associated with differentially methylated CpGs hypomethylated in responders at C7D1 vs C1D1. **(h)** Pathways enriched for genes associated with differentially methylated CpGs hypomethylated in responders compared to non-responders at C7D1. **(i)** Pathways enriched for genes associated with differentially methylated CpGs hypomethylated in responders at C7D22 vs C7D1. **(j)** Top 15 Metascape pathways associated with gene expression clusters at C1D1. Bar color indicates gene expression cluster shown in Figure 4h.

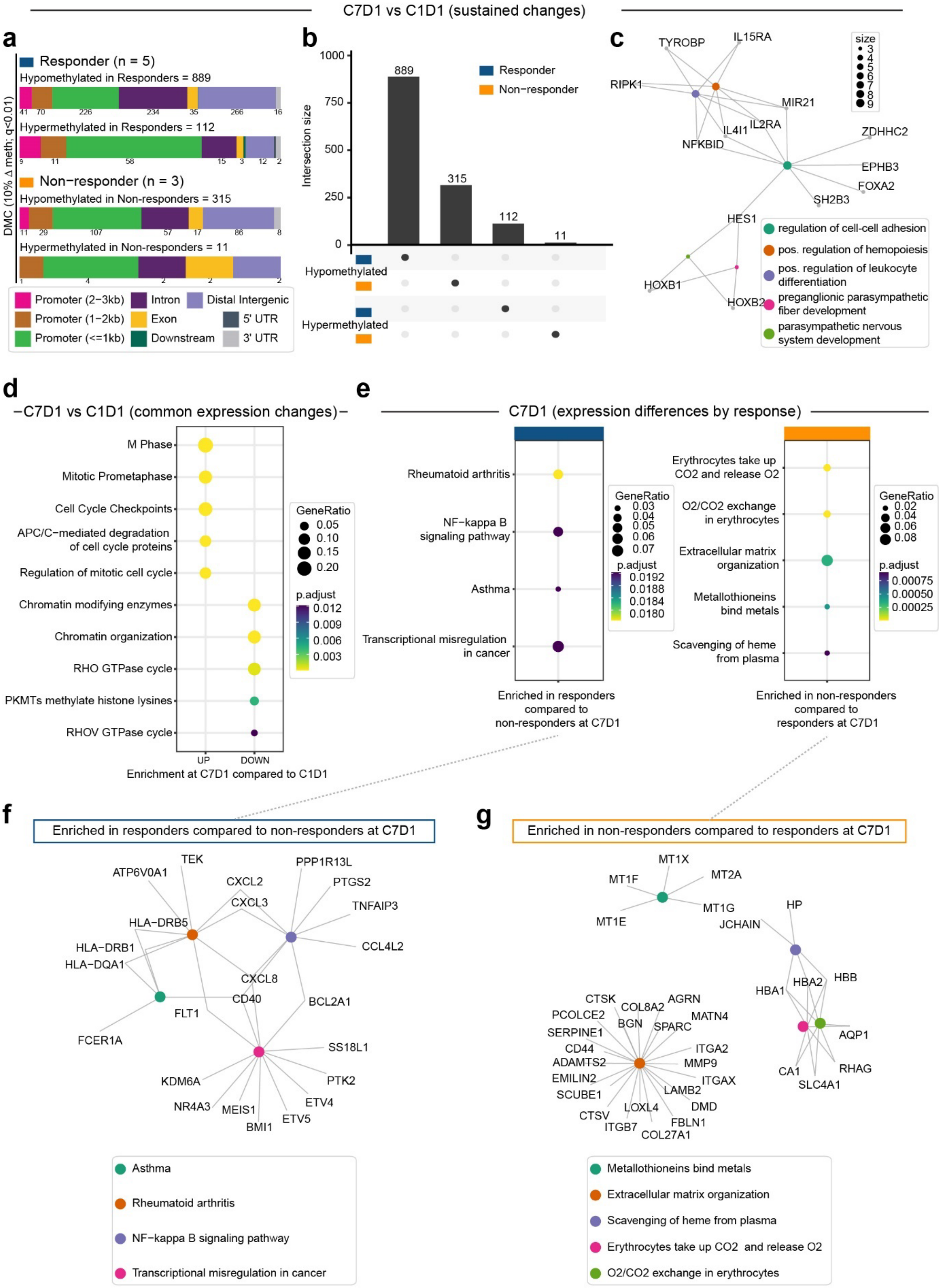

Supplementary Figure 6

Supplementary Figure 6: Differences in CpG methylation and gene expression in CD34+ HSPCs at C7D1 compared to C1D1

(a-c) Sustained changes in site-specific methylation. **(a)** Genomic distribution of CpG sites differentially methylated at C7D1 compared to C1D1. A total of 1239928 CpG were analysed in responders, and 1210117 CpG were analysed in non-responders. **(b)** Upset plot showing overlap of differentially methylated CpG sites between comparison groups. **(c)** Network diagram showing enriched pathways for genes mapping to CpGs which are hypomethylated in responders at C7D1 compared to C1D1 (n=889 CpGs). Network diagrams were created with clusterProfiler[3] using GO pathways[4]. CpGs were annotated to genes using HiChIP data from healthy human HSPC subsets[5]. **(d-g)** Changes in gene expression following 6 cycles of AZA treatment. **(d)** clusterProfiler[3] pathway[4] enrichment across all patients, irrespective of clinical response, at C7D1 vs C1D1. **(e)** clusterProfiler[3] pathway[4] enrichment in responders and non-responders at C7D1 vs C1D1. **(f-g)** Network diagram showing genes contributing to pathways enrichments shown in **(e)**. **(f)** Enriched in responders compared to non-responders at C7D1. **(g)** Enriched in non-responders compared to responders at C7D1.

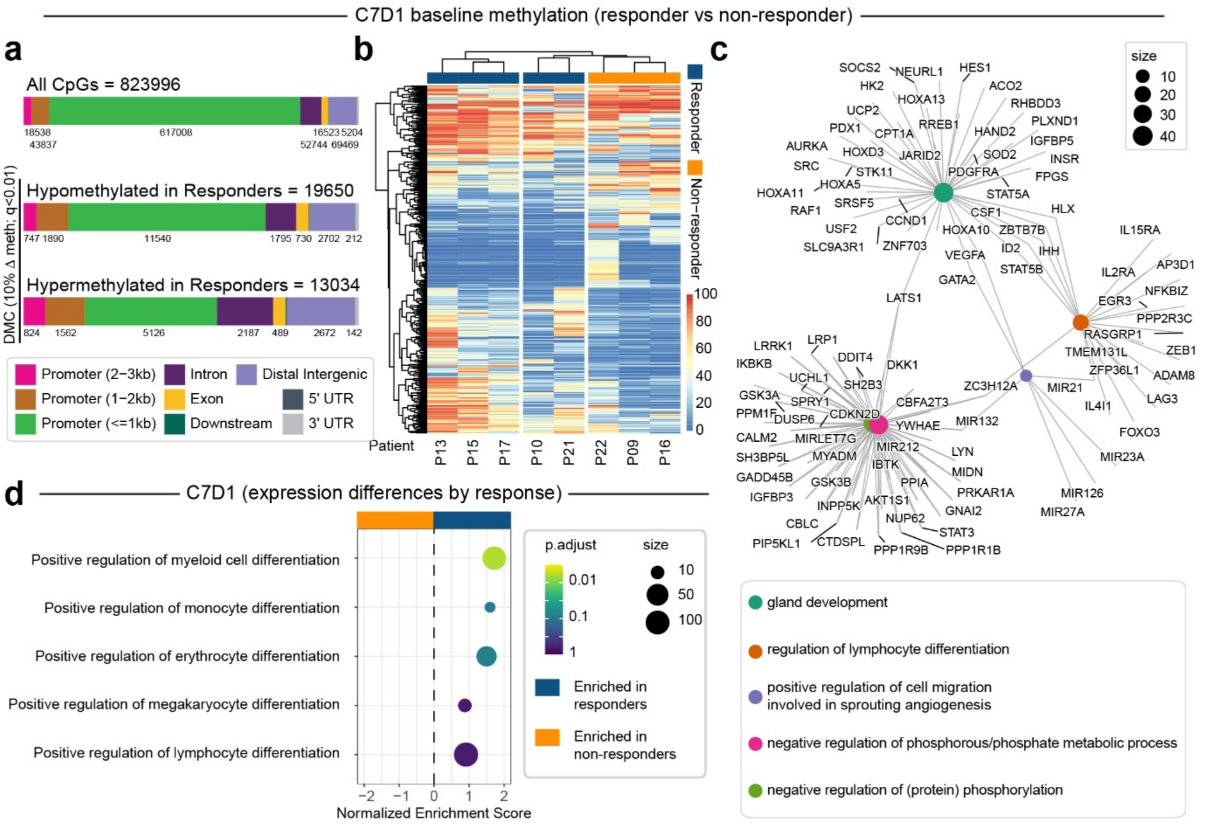

Supplementary Figure 7

Supplementary Figure 7: Differences in site-specific methylation in CD34+ HSPCs following six cycles of AZA treatment

(**a-c**) Comparison of responder (n=5) and non-responder (n=3) patients prior to commencing AZA therapy. **(a)** Upper bar shows genomic distribution of 823996 CpG sites with data in all samples at C7D1. Lower bars show genomic distribution of CpGs which were hypomethylated (n=19650) or hypermethylated (n=13034) in responders compared to non-responders. **(b)** Clustered heatmap showing differentially methylated regions across all patients, with clinical response as indicated. **(c)** Network diagram showing enriched pathways for genes mapping to CpGs which are hypomethylated in responders at C7D1. Network diagrams were created with clusterProfiler[3] using GO pathways[4]. CpGs were annotated to genes using HiChIP data from healthy human HSPC subsets [5]. **(d)** Gene expression following 6 cycles of AZA treatment. Normalized enrichment scores from FSGEA[6] analysis for pathways related to blood production in responders compared to non-responders at C1D1.

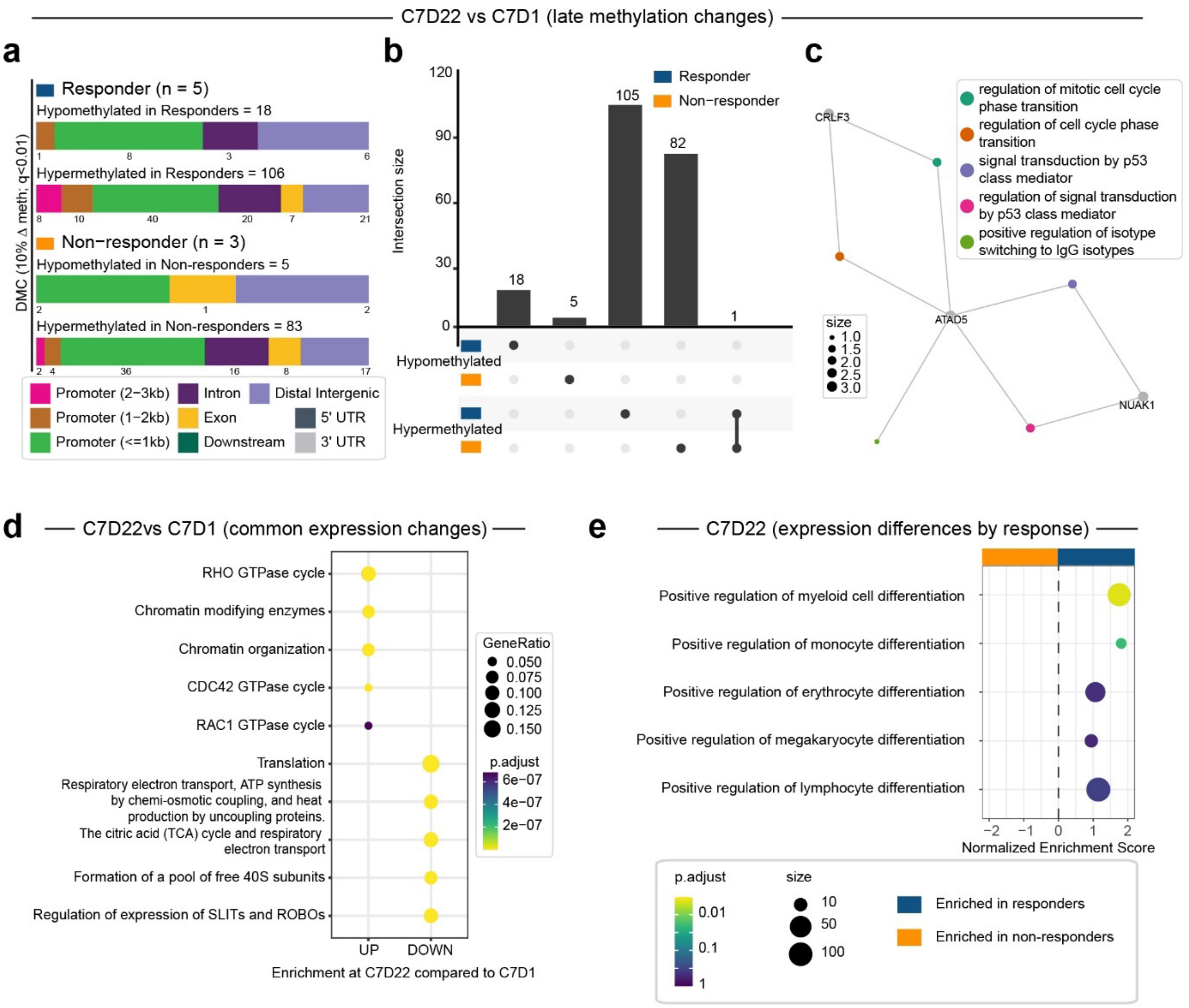

Supplementary Figure 8

Supplementary Figure 8: Differences in site-specific methylation in CD34+ HSPCs immediately following the first cycle of oral AZA

(a-c) Late changes in site-specific methylation. **(a)** Genomic distribution of CpG sites differentially methylated at C7D22 compared to C7D1. A total of 1144775 CpG were analysed in responders, and 1240814 CpG were analysed in non-responders. **(b)** Upset plot showing overlap of differentially methylated CpG sites between comparison groups. **(c)** Network diagram showing enriched pathways for genes mapping to CpGs which are hypomethylated in responders at C7D22 compared to C7D1 (n=18 CpGs). Network diagrams were created with clusterProfiler[3] using GO pathways[4]. CpGs were annotated to genes using HiChIP data from healthy human HSPC subsets [5]. **(d-e)** Changes in gene expression following cycle 7 of AZA treatment. **(d)** clusterProfiler[3] pathway[4] enrichment across all patients, irrespective of clinical response, at C7D22 vs C7D1. **(e)** Normalized enrichment scores for pathways related to blood production in responders compared to non-responders at C7D22.

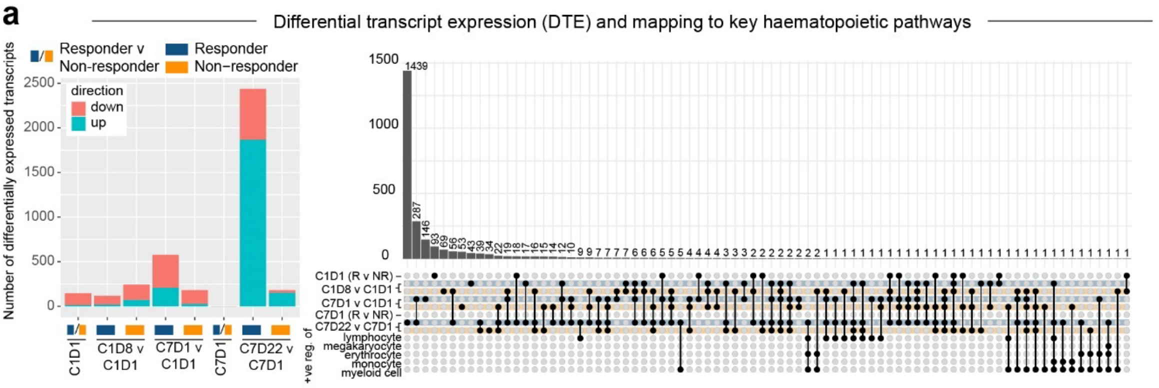

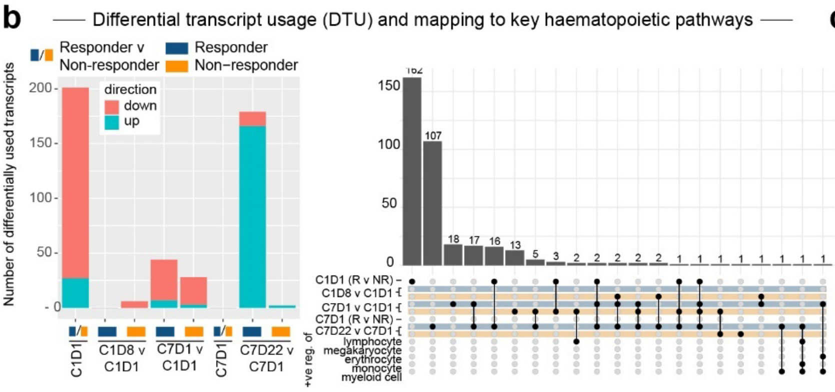

c Differential transcripts mapping to differentially methylated genes

C1D1 R v NR		DTE	***NBEAL2***, *KCNAB2, PTPRE, KLHDC7B, TYMP, NKTR*
		DTU	***NBEAL2***, ***SRSF5***, *KCNAB2, KLHDC7B, RNF220, NGDN, PIK3R5, TMEM259, CALM2, BCL2L1, PACS2, PSMG2, CD164*
C1D8 v C1D1		DTE	*ZNF609, RNASEH2A, KLF2, ARHGAP35, ZFP36L2, PPM1F, MN1, ST6GAL1, NRROS, H2AZ1*
		DTE	***SRSF5***, *RNASEH2A, H2AZ1, SRGN, HNRNPUL2, IRF2BPL, ZNF710, PPP1R15A, CCT4, PREX1, BCR, RREB1, INTS1*
C7D1 v C1D1		DTE	*NCOR2*
		DTE	*USF2*
C7D22 v C7D1		DTE	***NBEAL2***
		DTE	*RFX1*

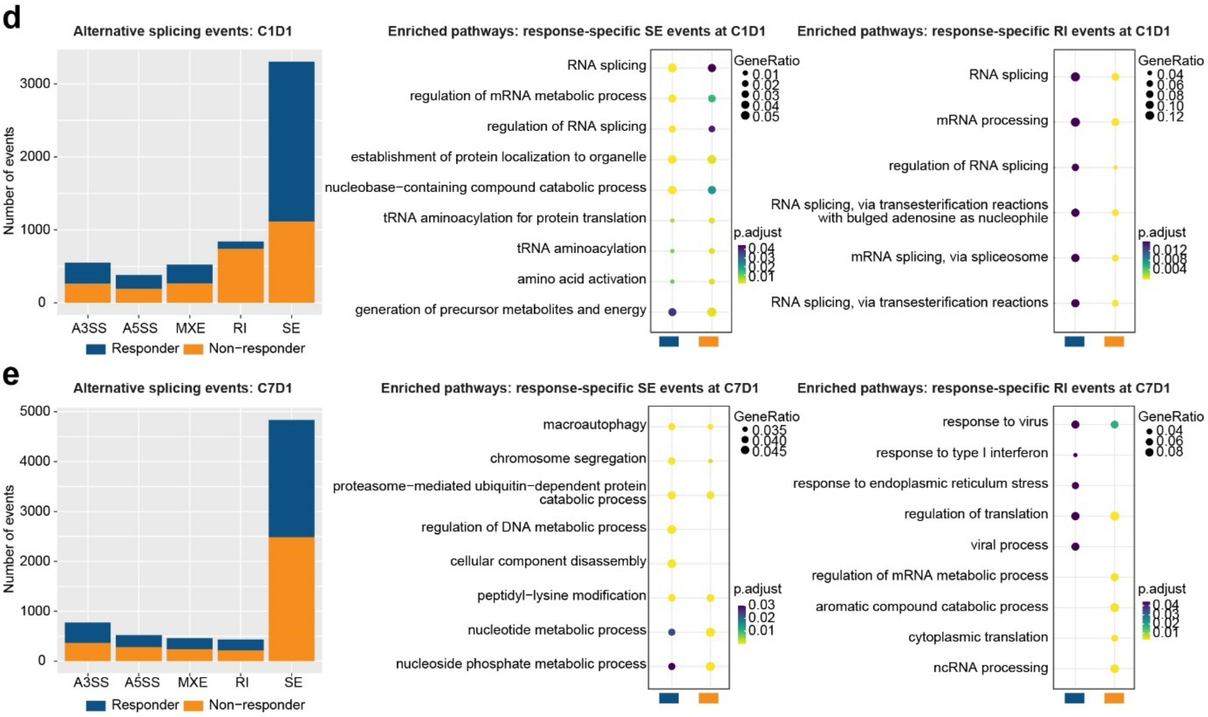

Supplementary Figure 9

Supplementary Figure 9: Longitudinal analysis of RNA isoform expression, use of alternate transcripts, and RNA splicing.

(a) Differential transcript expression, between response groups and across the course of treatment, and overlap with key haematopoietic pathways. **(b)** Differential transcript usage, between response groups and across the course of treatment, and overlap with key haematopoietic pathways. **(c)** Differentially expressed (DTE) or used (DTU) transcripts mapping the differentially methylated genes at the same comparison point. **(d)** RMATS analysis of alternate splicing events at C1D1, and clusterProfiler[3] pathway[4] enrichment associated with retained intron (RI) and skipped exon (SE) events. Blue bars indicate splicing events unique to responders while orange bars indicate splicing events unique to non-responders. **(e)** RMATS analysis of alternate splicing events at C7D1, and clusterProfiler[3] pathway[4] enrichment associated with retained intron (RI) and skipped exon (SE) events. Blue bars indicate splicing events unique to responders while orange bars indicate splicing events unique to non-responders.

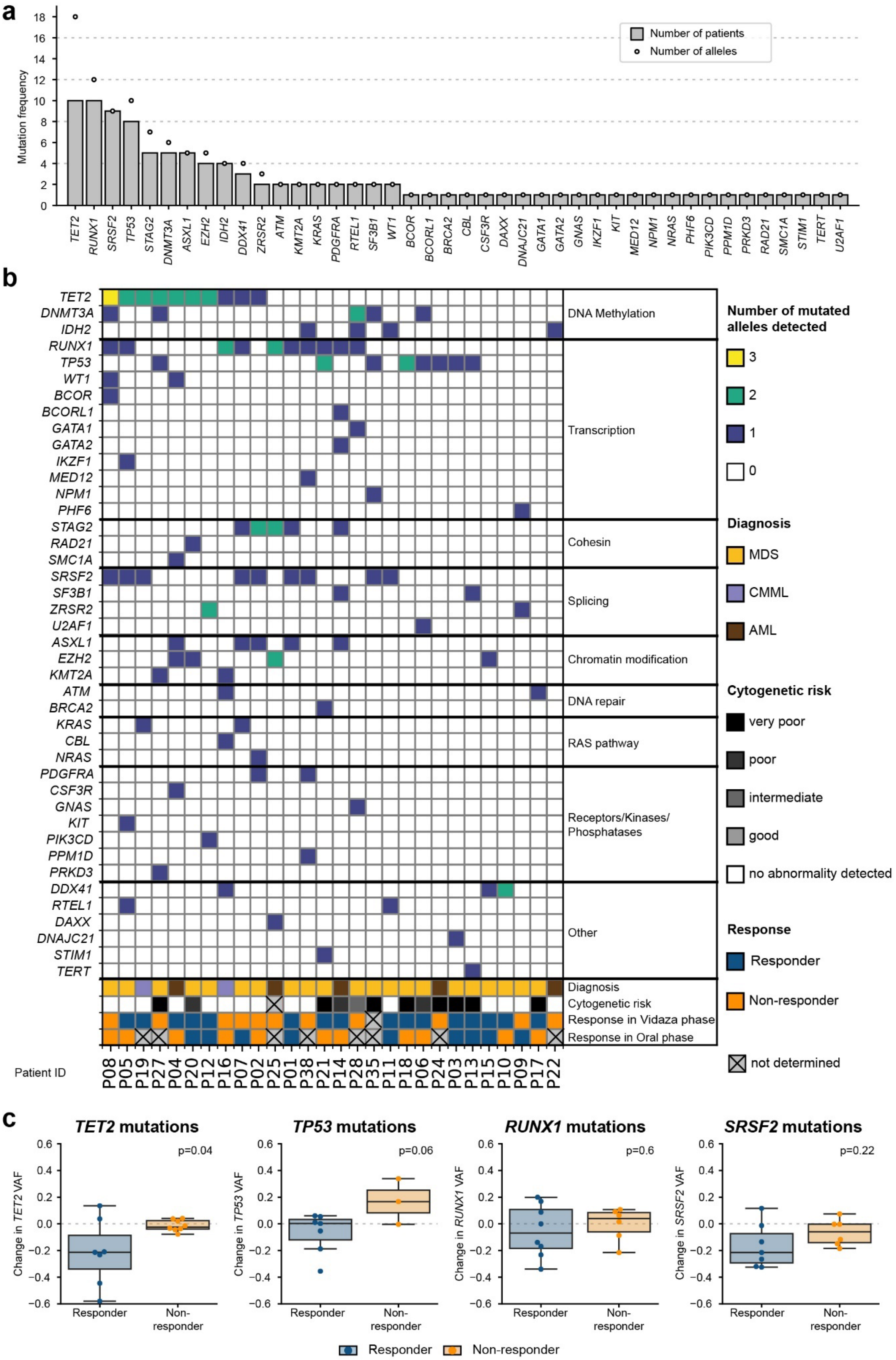

Supplementary Figure 10

Supplementary Figure 10: Variations in clonal composition in BM MNCs during HMA treatment

(a) Mutation frequency at diagnosis for 28 patients with available data. Grey bars show the number of patients with a mutation detected in the specified gene; open circles indicate the total number of mutated alleles detected in the cohort. **(b)** Overview of mutational profiles in each patient at diagnosis. For each patient, the number of mutated alleles corresponding to each gene is indicated, along with diagnosis, cytogenetic risk score, and response status. Genes are grouped by known function. Cytogenetic risk scores were classified as follows; "no cytogenetic abnormality detected", "good": normal karyotype; del(20q); del(5q); del(12p) or double including del(5q), "intermediate": +8; del(7q); i(17q); +19 or any other single or double independent clone, "poor": -7; inv(3)/t(3q)/ del(3q); double including -7/del(7q); or complex (3 abnormalities), "very poor": complex > 3 abnormalities. **(c)** Change in variant allele frequency and patient response status for genes with highest mutation frequency in the cohort. Graphs show combined injection and oral phases, with each data point showing VAF change and corresponding response status. Statistical comparisons were performed using two-sided ttest_ind (scipy.stats).

References

1. Unnikrishnan A*, et al.* Integrative Genomics Identifies the Molecular Basis of Resistance to Azacitidine Therapy in Myelodysplastic Syndromes. *Cell Rep* **20**, 572-585 (2017).

2. Graham SM, Vass JK, Holyoake TL, Graham GJ. Transcriptional analysis of quiescent and proliferating CD34+ human hemopoietic cells from normal and chronic myeloid leukemia sources. *Stem Cells* **25**, 3111-3120 (2007).

3. Yu G, Wang LG, Han Y, He QY. clusterProfiler: an R package for comparing biological themes among gene clusters. *OMICS* **16**, 284-287 (2012).

4. Milacic M*, et al.* The Reactome Pathway Knowledgebase 2024. *Nucleic Acids Res* **52**, D672-D678 (2024).

5. Subramanian S*, et al.* Genome-wide transcription factor-binding maps reveal cell-specific changes in the regulatory architecture of human HSPCs. *Blood* **142**, 1448-1462 (2023).

6. Korotkevich G, Sukhov V, Budin N, Shpak B, Artyomov MN, Sergushichev A. Fast gene set enrichment analysis. *bioRxiv*, 060012 (2021).
