# Supplementary material for: Clinical response to azacitidine in MDS is associated with distinct DNA methylation changes in HSPCs
Source: Nat Commun. 2025 May 13;16:4451. doi: 10.1038/s41467-025-59796-x (PMC12075701; doi:10.1038/s41467-025-59796-x)
Supplement: Supplementary file 3 — Description of Additional Supplementary Files [file 41467_2025_59796_MOESM3_ESM.pdf]

Description of Additional Supplementary Files

Supplementary Data 1

Description: Longitudinal variant allele frequencies. Variant alleles that are below detection limit at specific timepoints are indicated by “0”. Empty cells indicate that VAF analysis was not done for that patient/timepoint.
